# C-type lectin receptor-mediated immune recognition and response of the microbiota in the gut

**DOI:** 10.1093/gastro/goz028

**Published:** 2019-07-10

**Authors:** Tian-Hang Li, Ling Liu, Ya-Yi Hou, Su-Nan Shen, Ting-Ting Wang

**Affiliations:** 1 Immunology and Reproduction Biology Lab, Medical School of Nanjing University, State Key Laboratory of Pharmaceutical Biotechnology, Nanjing University, Nanjing, Jiangsu, P. R. China; 2 Jiangsu Key Laboratory of Molecular Medicine, Nanjing University, Nanjing, Jiangsu, P. R. China

**Keywords:** C-type lectin receptors (CLRs), microbiota, immunity, inflammatory bowel disease (IBD)

## Abstract

C-type lectin receptors (CLRs) are powerful pattern-recognition receptors that discern ‘self’ and ‘non-self’ in our body and protect us from invasive pathogens by mediating immune recognition and response. The gastrointestinal tract is very important for the maintenance of homeostasis; it is the largest shelter for the billions of microorganisms in the body and CLRs play a crucial regulatory role in this system. This study focuses on several CLRs, including Dectin-1, Dectin-2, Dectin-3 and Mincle. We summarize the roles of CLRs in maintaining gastrointestinal immune-system homeostasis, especially their functions in mediating immune recognition and responses in the gut, discuss their relationships to some diseases, highlight the significance of CLR-mediated sensing of microbial and non-microbial compounds in the gut immune system and identify new therapeutic targets.

## Introduction

In our bodies, the intestinal environment provides a shelter for an enormous number of microorganisms; bacteria comprise up to 60% of the dry mass of feces. Other types of intestinal microorganisms include fungi, archaea and viruses. Compared with that of bacteria, the incidence of pathogenic fungi infections is relatively low; therefore, we might easily undervalue their potential threat to our health. In fact, several studies have identified that the gut fungal microbiota also exert important functions in the gut [1, 2]. In contrast to the commensal bacterial community, the fungal community is unstable and prone to higher variability in the gut [3]. Whereas recent studies have found that the fungal microbiota is more stable than originally thought, it is significantly altered in certain intestinal diseases, such as inflammatory bowel disease (IBD) [4, 5]. In the gastrointestinal tract of healthy individuals, only a small number of fungal species, including *Candida*, *Saccharomyces* and *Aspergillus*, colonize and grow steadily [6–8]. Dysbiosis of the gut bacteria has been studied for several decades and this specific term describes a perturbed condition involving alterations in the gut flora that can be both result from and cause various pathologies [9].

The gastrointestinal tract is the longest visceral cavity in the body, with a length of approximately 16 feet and a massive absorptive area due to its microvilli [10]. As it is in direct contact with food-derived antigens, the commensal gut microbiota and potential invasive pathogens that enter through the diet, the gut immune system has developed sophisticated mechanisms for sensing pathogenic and non-pathogenic microorganisms and for initiating an immediate response under threats from pathogens [1]. The gut immune system is necessary for homeostasis maintenance in the gut microenvironment, as it maintains a delicate balance among bacteria, fungi and the gut epithelium.

The recognition of fungal pathogens and initiation of the subsequent antifungal immune responses in the gut are mostly mediated by members of the C-type lectin receptor (CLR) family. CLRs play critical roles in gastrointestinal antifungal immunity by serving as pattern-recognition receptors (PRRs). Some key members include Dectin-1, Dectin-2, Dectin-3 and Mincle. These CLRs recognize pathogen-associated molecular patterns (PAMPs) expressed on certain fungal cell walls and induce innate and adaptive immune responses [11].

IBD is a common and complex intestinal disease that is clearly associated with intestinal dysbiosis [12, 13]. IBD has two main clinical manifestations: Crohn’s disease (CD) and ulcerative colitis (UC) [14]. Both bacteria and fungi can perturb harmonious host–microbe symbiosis and immune homeostasis. Therefore, CLRs, as innate immune receptors, may play vital roles in IBD progression. There is also a link between cancer and the gut microbiota [15]. Since cancer susceptibility and severity are associated with inflammation [16], there may be a relationship between oncogenesis, tumor progression and antifungal immunity [17] because the antifungal immunity is mainly mediated by CLRs.

The central roles that CLRs play in promoting immunity to fungal pathogens are also shared with other PRRs such as Toll-like receptors (TLRs) [18–20], suggesting that it may be possible to target some of these receptors, such as Toll-like receptor 1 or Toll-like receptor 9, to induce more efficient immune responses. The collaborations between CLRs and other PRRs might form the basis for new combinative approaches for fighting gastrointestinal disorders and related diseases.

## Mucosal immunity to the gut microbiota

The gastrointestinal microbiota plays a critical role in shaping host immunity. In return, the host immune system uses a variety of mechanisms to maintain a harmonious symbiosis [21]. This process involves the induction of protective immune responses to pathogens as well as the regulatory immunity required for ongoing tolerance of resident microbiota during homeostasis and the immune responses during dysbiosis [8, 22]. Our knowledge about mucosal immunity to the gastrointestinal microbiota mostly came from work performed under conditions of pathogen infection, while the functions of the immune system and the microbiota during homeostasis or dysbiosis remain unclear. In this section, we discuss the regulatory roles of the gastrointestinal immune system in the maintenance of homeostasis with a focus on the fungal community.

Alterations in the intestinal mycobiota may also be associated with the susceptibility and severity of various diseases. Beneficial fungi and pathogenic fungi can be identified in the gastrointestinal tract during homeostasis and dysbiosis. The beneficial fungi are usually defined as harmless commensals living in the gut and the appropriate proportion of the beneficial fungi remains stable to maintain a delicate balance. In contrast, pathogenic fungi are found to be increasing in many diseases and influence the progression of the disease. *Saccharomyces cerevisiae* is thought to be beneficial to gut microbial ecology and has probiotic effects, while, if co-morbidities are present, *S. cerevisiae* may give rise to the increasing incidence of *Saccharomyces* fungemia [6]. This example suggests that the pathogenic fungi and beneficial ones can interconvert into each other. Many factors could contribute to fungal dysbiosis, including use of antibiotics, diet and genetic factors. Compared with intestinal fungal infections, which are relatively rare, fungal dysbiosis occurs more frequently in the gastrointestinal tract. For example, *Candida albicans* is a well-known commensal in the gut of healthy individuals; however, it can also be pathogenic in the gastrointestinal tract. A shift from a normal commensal state to a dangerous pathogen state can be caused by antibiotic use. One study found that treatment with oral antibiotics can lead to overgrowth of *Candida* populations in the gut of people and mice [23]. Furthermore, the symbiosis between bacteria and fungi may also affect how the host immune responses control fungal colonization and growth. Mice colonized with *Bacteroides thetaiotamicron* are more resistant to the colonization of *C.* *albicans* than the germ-free mice via production of anti-microbial peptide LL-37 [24].

PRRs are often activated during infection; however, CLRs also exert their important host-protective functions during dysbiosis. *C. albicans* is a common commensal microbiota in the gut. Dectin-2 knockout mice were more susceptible to *C.* *albicans*, indicating the role of Dectin-2 in recognizing the yeast in its commensal state and in suppressing the overgrowth of this potentially pathogenic microorganism [25]. Our understanding of normal fungal diversity and alterations therein based on diet or other environmental factors is still at an early stage. Nevertheless, an increasing number of studies suggest that the diversity in the intestinal fungal microbiota is skewed in many diseases such as autism, obesity and asthma [26].

During homeostasis, commensal fungi exert many of their physiological effects in the gastrointestinal tract by facilitating nutrient absorption and digestion via production of enzymes and vitamins. A study found that *Bacteroides thetaiotaomicron*, a prominent member of the gastrointestinal microbiota, uses yeast mannan as its food source via a selfish mechanism, indicating an additional way in which the fungal microbiota could participate in gastrointestinal physiology [27]. Since CLRs are mainly expressed on the immune cells in the lamina propria, fungal molecules rarely reach CLRs during homeostasis due to the normal regeneration capability and integrity of the mucosal surface epithelium.

## Structure and activation of CLRs

**Figure 1.**
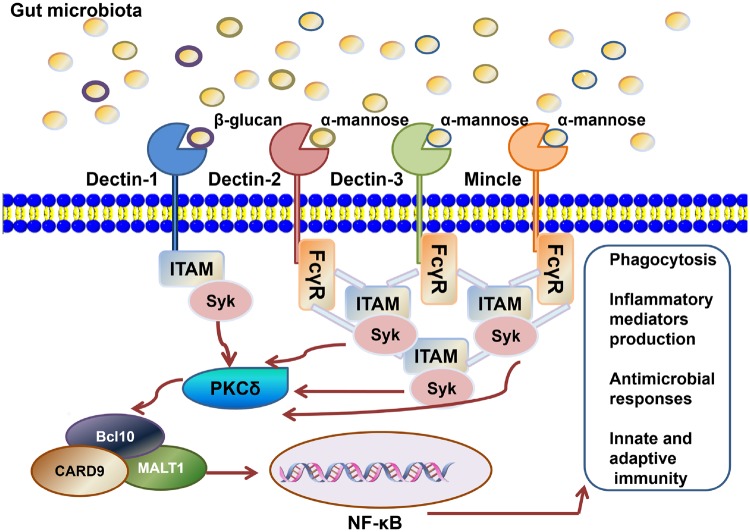
C-type lectin receptors (CLRs) are transmembrane pattern-recognition receptors (PRRs) expressed on myeloid cells. Dectin-1, Dectin-2, Dectin-3 and Mincle induce intracellular signaling via their immunoreceptor tyrosine-based activation motif (ITAM), which can recruit spleen tyrosine kinase (Syk). Dectin-1 directly recruits Syk while Dectin-2, Mincle and Dectin-3 indirectly activate Syk via the Fcγ receptor (FcγR) adaptor chain. Downstream signaling via protein-kinase C (PKCδ) activates the CARD9–Bcl-10–MALT1 complex, which then activates nuclear factor-κB (NF-κB) to induce the production of various inflammatory mediators. Dectin-2, Mincle and Dectin-3 can form dimers with each other to work synergistically. CLRs can induce and repress the production of various cytokines and chemokines. Furthermore, CLRs mediate both phagocytosis and innate and adaptive responses.

CLRs are the main PRRs that mediate the antifungal immune responses in the gut. They can be differentiated from other PRRs by the C-type lectin domain in their extracellular region (Figure 1). The PAMPs that CLRs recognize are the carbohydrates on the pathogen surface [28]. The intestinal mucosal barrier is the first line of defense in the protection of our gut against infection [4]. However, many factors can damage this barrier, which can then allow invasion by intestinal pathogens, such as a lack of or over activation of certain immune responses due to disruption of the gut flora [29]. Furthermore, microfold cells (M cells) are an important contributing factor because they serve as a portal, through which the pathogen can cross the barrier to induce subsequent immune responses in the intestinal phagocytes of the lamina propria [30]. During this process, CLRs play various important roles of mediating immune recognition and response.

### Dectin-1

Dectin-1 is primarily expressed by myeloid cells and serves as the predominant PRR for mediating antifungal immunity in the gut. β-glucans are the major PAMPs present within the fungal cell walls that are recognized by Dectin-1. Dectin-1 expression was first identified in dendritic cells (DCs) and it was later shown to be widely expressed in various cell types, including monocytes, macrophages and neutrophils [31].

Dectin-1 is a 28-kDa type II membrane protein. An extracellular C-type lectin-like domain is connected by a stalk to a transmembrane region and this stalk contains a special region. The length of the stalk varies to facilitate β-glucan recognition and a longer length induces better binding efficiency. This region is defined as the carbohydrate-recognition domain (CRD). Trp221 and His223 are the two crucial residues for β-glucan binding. The Dectin-1 cytoplasmic tail contains an immunoreceptor tyrosine-based activation motif (ITAM)-like motif, which is involved in the activation of intracellular signaling. In contrast to classical CLRs, Dectin-1 is atypical due to its metal-ion-independent carbohydrate recognition [32, 33].

Upon ligand binding, Dectin-1 is a tyrosine phosphorylated by Src kinase. This phosphorylation leads to docking and activation of the tandem SH2-domain-containing protein spleen tyrosine kinase (Syk). Through the PKCδ- caspase recruitment domain family, member-9 (CARD9)–B-cell lymophoma-10 (Bcl-10)–mucosa-associated lymphoid tissue lymphoma translocation protein 1 (MALT1) axis, nuclear factor-κB (NF-κB) is finally activated, inducing an intracellular signaling cascade that mediates various cell-specific responses, such as reductions in the levels of various cytokines and chemokines, the respiratory burst and phagocytosis-mediated ligand uptake. The induction of adaptive immune responses, particularly the T helper 1 (Th1) and T helper 17 (Th17) cell responses, is also directed by Dectin-1. Furthermore, Dectin-1 collaborates with other PRRs, such as Toll-like receptor 4 (TLR4) and Dectin-2 [34, 35].

As discussed above, β-glucan recognition by Dectin-1 is the start of phagocytosis and the initiation of protective immune responses. The immunological potency of β-glucans comes from their molecular mass, spatial stereoscopic structure and degree of branching [36]. The natural 3D structure of β-glucans depends on these three characteristics. β-Glucan species of longer lengths cannot form a crystal lattice in complex with Dectin-1 [37]. However, if the length of the β-glucan is too short (less than 10- or 11-mer), the binding will be weak. Furthermore, Dectin-1 oligomerization also increases the affinity for β-glucans. Additionally, whether the binding affinity of Dectin-1 is related to the type of β-glucan remains unclear [38]. The triple helix conformation of β-glucan enhances its biological functions related to its antitumor activity [39]. DCs and macrophages are activated and initiate phagocytosis after recognition of β-glucan by Dectin-1. However, if the β-glucan is fragmented, DCs and macrophages cannot be activated [40].

### Dectin-2

Dectin-2 is a C-type lectin-like receptor that is expressed in myeloid cells and it is mainly expressed on DCs, monocytes and macrophages. Unlike Dectin-1, which recognizes β-glucans, Dectin-2 is a PRR that binds mannose and its CRD contains a glutamic acid-proline-asparagine (EPN) motif, which is Ca^2+^-dependent. Unlike Dectin-1, which recruits and activates Syk directly, Dectin-2 interacts with an Fc receptor γ subunit (FcRγ) on the cell surface that contains a Syk-interacting immunotyrosine activation motif. A range of signaling pathways is initiated that ultimately lead to NF-κB activation, which then promotes the production of various cytokines and mediates the subsequent innate and adaptive immune responses. Furthermore, Dectin-2 and other CLRs (including Dectin-3) can functionally collaborate. Dectin-2 and Dectin-3 interact in a heterodimeric form to mediate the defense against fungal infection [41, 42].

### Mincle

Mincle is a member of the Dectin-2 family of CLRs and it is mainly expressed on activated macrophages. Mincle was first discovered to be an important player in gastrointestinal anti-microbial immunity based on its ability to bind a variety of PAMPs derived from pathogenic fungal microbiota, including α-mannose, lipidic species and some endogenous self-ligands, such as Sin3A-associated protein 130 (SAP130), which is a product of dead cells. Dectin-2 is a type II transmembrane protein with a short cytoplasmic tail and its extracellular domain is responsible for ligand binding. Like that of Dectin-2, Mincle-dependent signaling also occurs via FcRγ culminating in a pathway involving Syk and the CARD9–Bcl-10–MALT1 complex to activate NF-κB, which then leads to several pro-inflammatory responses and adaptive immune responses against pathogens [43, 44].

### Dectin-3

Unlike the other three receptors mentioned above, the characteristics and functions of Dectin-3 remain obscure. Dectin-3, known also as *Mcl/Clecsf8/Clec4d*, is a myeloid cell-specific CLR family member. The PAMPs it recognizes are mainly mycobacterial trehalose 6, 6-dimycolate (TDM) as well as α-mannans that exist in the fungal cell wall. Unlike Dectin-1, which contains an ITAM in its cytoplasmic region, Dectin-3 has no signaling motif in its cytoplasmic domains; instead, it signals downstream via the ITAM-containing adaptor molecule FcRγ. The CRD of Dectin-3 is Ca2^+^-independent for carbohydrate recognition. Dectin-3 may use a signaling pathway similar to those of the other three CLRs to mediate intestinal antifungal immunity. More studies are required to gain more detailed and precise information about its structure and activation [42, 45, 46].

## CLRs-mediated immune recognition of the gut MICROBIOTA

Recognition of invading pathogens by the host is the first step and a key stage in initiating an effective immune response after gastrointestinal fungal infection [47]. Efficient immune recognition of microorganisms initiates the crosstalk between the host and gut microbiota. And this process in turn helps to maintain gastrointestinal homeostasis via tolerance of the normal commensal microorganisms.

CLRs play key roles in immunity to fungal pathogens in the gut microbiota. These receptors bind to almost all the pathogenic fungi found in the gut. The recognition specificity of the CLRs is largely based on different carbohydrate structures expressed by different fungal species as well as various morphological forms of the same organism [48]. Different fungal PAMPs can be exposed because the morphological forms of the gut fungi are constantly alternating between the yeast and hyphae state [49]. For example, the cell wall of *C.* *albicans* mainly contains carbohydrate polymers and glycoproteins. The components of the cell wall comprise mainly chitin, β-1, 3- and β-1, 6-glucans and O- and N-linked mannan. In *C.* *albicans*, the β-1, 3- and β-1, 6-glucans are shielded by mannoproteins, while, in the budding yeast and hyphae forms, they are expressed on the surface of the cell wall. In contrast, the cell wall of *Aspergillus* predominantly contains chitin, α-glucans, β-1, 3-glucans and β-1, 4-glucans, and galactomannans [50].

As shown in Table 1, different CLRs recognize and bind to the various PAMPs expressed on the fungi and mediate the downstream immune responses. Dectin-1, as discussed above, is the primary CLR in the gut that recognizes β-1, 3-glucan chains. Therefore, both *C.* *albicans* and *Aspergillus fumigates* can be recognized by Dectin-1. Dectin-1 can only recognize particulate β-glucans to induce cytokines and initiate internalization of the fungus [50]. A recent study found that, upon infection, Dectin-1 can modulate interleukin-1 receptor-associated kinase 1 (Irak1) and receptor-interacting protein 2 (Rip2), which are the key adaptor proteins in the TLR and Nod-like receptor signaling pathways, respectively [51]. This finding indicates that *A. fumigatus* recognition by Dectin-1 may involve interplay with other PRRs in the gut. Human primary myeloid DCs can also interact with *A. fumigates* via Dectin-1 [52]. As Dectin-2 recognizes mannans, the mycobiota that Dectin-2 recognizes are *C. albicans*, *Candida glabrata* and *Cryptococcus neoformans* [53]. Dectin-2 can cooperate with Dectin-3 by forming heterodimers to recognize *C.* *albicans* [45]. Dectin-2 is also involved in *A. fumigatus* recognition and its expression is restricted to macrophages [54]. A recent study found that Dectin-2 participates in the recognition of *Pneumocystis*, which is a potential gut pathogen [55]. *Cryptococcus* *neoformans* is a yeast-type opportunistic fungal pathogen that is normally associated with the respiratory system and can damage the central nervous system. Dectin-2 also participates in *C. neoformans* recognition and mediates the antifungal immunity against this pathogen [56]. Although the binding ligand for Mincle in the gastrointestinal fungi remains unclear, Mincle recognizes *C. albicans* and mediates the macrophage-dependent immune response via TNF-α production [57]. It is interesting that Mincle recognizes not only fungi, but also *Mycobacterium* via the TDM glycolipid (also known as cord factor) cell-wall component [58].

**Table 1.** C-type lectin receptors and their respective PAMPs and microbiota involved in gastrointestinal immunity

CLR	Ligand(s)	PAMP	Microbiota	References
Dectin-1	β-1, 3-glucan	β-1, 3-glucan	*Candida albicans*	[50–52]
			*Aspergillus fumigatus*	
Dectin-2	α-mannan	α-mannan	*Candida albicans*	[45, 53–56]
			*Candida glabrata*	
			*Cryptococcus neoformans Aspergillus fumigatus*	
Dectin-3	α-mannose	α-mannose	*Candida tropicalis*	[46, 62–64]
	mycobacterial trehalose6, 6-dimycolate			
Mincle	Glyceroglycolipid	α-mannose	*Candida albicans*	[57–59]
	mannosyl fatty acids			
	α-mannose			

CLR, C-type lectin receptor; PAMP, pathogen-associated molecular pattern.

Mincle is also associated with recognition of other bacteria such as *Listeria monocytogenes*, *Klebsiella pneumonia*, *Streptococcus pneumonia*, *Pneumocystis pneumonia* and *Escherichia coli*, and all of these microorganisms are thought to be associated with CD [59]. These clues indicate that we may pay more attention to the possible relationship between Mincle and the pathogenesis of IBD [60]. Furthermore, Mincle can recognize various lipid components released from both host cells and microorganisms released upon cell death [44, 61]. In relation to Dectin-3, we found that the development of dextran sulfate sodium (DSS)-induced colitis can be promoted by Dectin-3 deficiency in mouse models challenged with *Candida tropicalis* [46], supporting its role in fungal recognition in the gut. A recent study also found that Dectin-3 expression is involved in the antifungal immune response by plasmacytoid DCs to *C. neoformans* infection *in vitro* [62]. Another study compared the responses to pulmonary *cryptococcal* infection between Dectin-3 knockout mice and wild-type mice, and found that the murine immune responses to *C. neoformans* infection did not necessarily require Dectin-3 [63]. Dectin-3 can also recognize TDM and induce Mincle expression upon *Mycobacterium* infection via CARD9–Bcl-10–MALT1-dependent NF-κB activation and the increased Mincle expression can enhance the ability of host innate immune system to sense *Mycobacterium* infection [64].

## CLRs-mediated immune response against the gut microbiota

**Figure 2.**
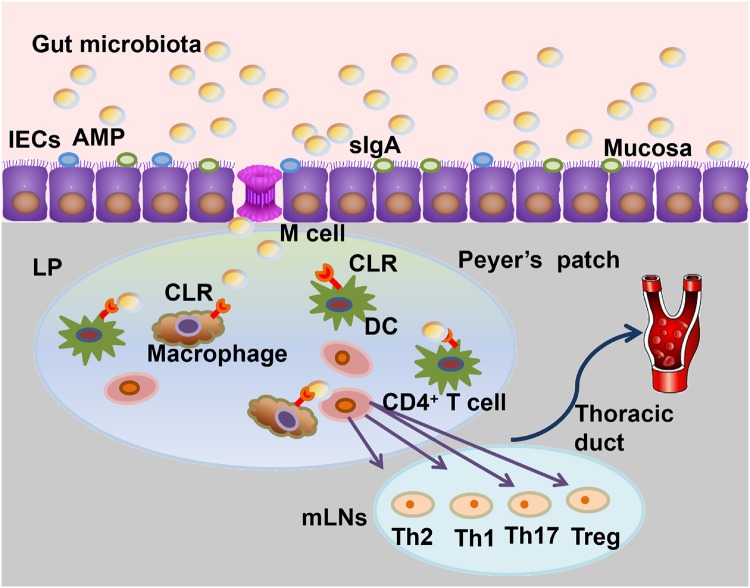
The intestinal mucosa serves as a physical barrier to defend against pathogen invasion via mucus and anti-microbial peptides (AMPs) production by the intestinal epithelial cells, such as Paneth cells. The composition and translocation of the gut microbiota are controlled by the intestinal epithelial cells (IECs). Microfold cells (M cells) are a potential point of entry for microorganisms. Depending on the type of invading microbiota, the C-type lectin receptors (CLRs) expressed on the immune cells in the lamina propria (LP) function as pattern-recognition receptors (PRRs) to recognize the microbes and promote their degradation by phagocytes. Antigen presentation by dendritic cells (DCs) and macrophages stimulates the naïve T cells to differentiate into T helper (Th) cells and regulatory T (Treg) cells, which then enter the blood circulation via the thoracic duct to initiate the adaptive immune response. sIgA, secretory immunoglobulin A; mLNs, mesenteric lymph nodes.

As discussed above, CLRs are the key PRRs in the intestinal microenvironment that mediate antifungal immunity. Both the innate and adaptive immune responses are induced and modulated by these receptors to resist infection and maintain homeostasis of the gut microbiota. The CLR-mediated innate immune responses mainly include fungal binding and phagocytosis, induction of antifungal effector mechanisms and the production of a variety of soluble mediators, such as cytokines, chemokines and inflammatory lipids [65]. The expression of these soluble mediators can contribute to the host adaptive immunity, which mainly involves direction and modulation of the Th1, Th2, Th17 and regulatory T cell (Treg) responses [48] (Figure 2).

It is commonly accepted that the intestinal mucosal barrier is indispensable for protection against invasion of pathogens from the gut microbiota and for limiting their overgrowth or translocation [21]. The intestinal mucosa barrier not only serves as a mechanical defense barrier, but it also contains many types of mucin glycoproteins, anti-microbial peptides and secreted immunoglobulins (sIgA and IgG), all of which are involved in protecting the gastrointestinal mucosa of the host. Furthermore, M cells, which are located on the gut-associated lymphoid tissue of Peyer's patches and the mucosa-associated lymphoid tissue of other parts of the gastrointestinal tract lacking mucin, can serve as a portal. Pathogens, particularly *C. albicans*, can cross the barrier through this portal [66]. As discussed above, CLRs expressed on myeloid cells recognize pathogens in the gut and mediate the downstream immune responses. Many pro-inflammatory cytokines and chemokines, such as IL-1α/β, IL-6, G-CSF, GM-CSF, TNF-a, IL-8 and CCL20, can be produced following *C. albicans* recognition by epithelial cells [67]. One study found that sIgA delivery via M cells depends on Dectin-1 along with the Siglec-5 receptor, suggesting a role for Dectin-1 in the first line of defense in the gut immune response [68]. More recently, Dectin-3 deficiency was found to promote the development of colitis induced by severe damage to colon epithelial cells and impaired tissue repair in DSS-induced colitis models, indicating that CLRs may have additional roles in protecting the intestinal mucosa [46]. Intestinal epithelial cells (IECs) appear to be able to recruit lymphoid and myeloid cells to the mucosal layer [69], but whether this activity is related to CLRs remains unclear.

The intestinal phagocytes, which are mainly located in the lamina propria, play the central role in the innate immune response against invasive pathogens once they cross the intestinal mucosal barrier [65, 70]. Phagocytes include macrophages, DCs, lymphoid cells, leukocytes and mast cells. These immune cells participate in the response to the pathogenic microorganisms and contribute to the tolerance of the normal intestinal flora. As discussed above, after being recognized by CLRs expressed on phagocytes, invasive microorganisms are internalized via the actin-dependent process of phagocytosis [71]. In DCs, Dectin-1 triggers phagocytosis via its cytoplasmic ITAM-like motif in a process that requires Syk [72]. After phagosome maturation, oxidative and non-oxidative mechanisms are used synergistically by phagocytes to kill internalized or extracellular fungi. The oxidative mechanisms include the respiratory burst and reactive nitrogen intermediates; the non-oxidative mechanism mainly depends on the production of anti-microbial peptides (AMPs), hydrolases and nutrient limitation [65]. Different phagocytes have various abilities to kill fungi or to limit their growth, depending on the specific fungal species. For example, neutrophils are the main cells that kill *C. albicans*, while DCs are more potent against *Histoplasma capsulatum* [73, 74].

Upon recognition of pathogens by CLRs, Syk/CARD9 induction activates NF-κB signaling, ultimately leading to the secretion of pro-inflammatory cytokines, including GM-CSF, TNF, MIP-2, IL-2, IL-10, IL-6, IL-23 and IL-1β. This signaling network initiates the differentiation of naïve T cells into Th cell lineages, including Th1, Th2, Th17 and Tregs, which are critical for antifungal immunity. Antigen presentation by the antigen-presenting cells (mainly DCs) to T cells normally occurs in the lamina propria [66] and Th17 differentiation is mainly promoted by induction of IL-1β, IL-6 and IL-23, which drives IL-17 and IL-22 production [75]. Neutrophil recruitment, epithelial AMP production and mucosal antifungal protection can also be driven by these cytokines [65]. However, aberrant Th17 responses may cause unexpected pathogenic hyper-inflammatory disorders that can also be tightly controlled by IL-1β production that is induced due to Dectin-1 activation [76]. The Th1 response, which depends on IL-12p70 and can also be triggered via a Dectin-1-directed Syk-Raf1 cooperative signaling pathway, can be shifted to a Th2 cell response by inhibition of the Raf1 or IRF1 signaling pathways [11]. Cooperation between the Dectin-1-induced signaling pathways is essential for the Th cell response that shapes the antifungal adaptive immune response.

Unlike Dectin-1, which is capable of self-activation, Dectin-2 is activated via its association with FcRγ. Dectin-2 expressed on DCs recognizes fungal hyphae [77] (which consists mainly of α-mannoses) to promote NF-κB activation via Syk recruitment and subsequent activation of the CARD9–Bcl-10–MALT1 complex. The cytokines induced through this process, including IL-6, IL-23, IL-12 and IL-1β, lead to the induction of Th17 responses. A range of pro-inflammatory cytokines, including TNF, IL-6 and IL-23, as well as several anti-inflammatory cytokines, such as IL-2 and IL-10, can also be induced by Dectin-2 to promote Th17 responses [74, 78]. Depending on the DC subset and specific ligands, different signaling pathways can be activated via Mincle’s association with FcRγ (similar to Dectin-2) to mediate the adaptive antifungal immune response. In human DCs, Mincle triggers the Syk-dependent CARD9–BCL-10–MALT1 complex pathway, which then results in phosphoinositide 3-kinase (PI3K)- and PKB (protein-kinase B)-dependent activation of the E3 ubiquitin ligase. The activation of the E3 ubiquitin ligase will suppress IL-12p70 production to skew Th cell differentiation in favor of Th2 cell responses [79]. Furthermore, Treg cells can help regulate the immune response during intestinal infection. In mouse models, the numbers of CD4^+^CD25^+^ cells and Foxp3 Tregs in the mesenteric lymph nodes (mLNs) and stomachs of infected mice are dramatically increased during intestinal *C.* *albicans* infection. CLR-mediated T cell differentiation towards Tregs also plays important roles in homeostasis in the intestinal environment [66].

## CLRs-mediated antifungal immunity and related diseases

The prevalence of IBD is expanding globally, now affecting over 1 million individuals in the USA and over 2.5 million individuals in Europe, as well as a significantly increased number of people in Asia, South America and the Middle East [80, 81]. IBD pathogenesis has a genetic basis and involves the immune responses activated by various environmental factors [14]. In the pathogenesis of IBD, immune tolerance can be reduced by the microbes, which can then result in activation of inappropriate pathways intended to protect the host against pathogen invasion [82]. Most studies on microbe in the gut focus on the analysis of bacteria [83–85]. Recently, the role of fungi has also attracted our attention. By using ITS2 sequencing, the fungal community in the feces of 235 patients with IBD was identified. There is a skew in the fungal microbiota of the IBD patients, with an increased *Basidiomycota/Ascomycota* ratio, a decreased proportion of *S.* *cerevisiae* and an increased proportion of *C. albicans* compared with the microbiota of healthy subjects. It is interesting that, in the gut environment of IBD, fungal growth is favored over bacteria growth [12]. However, after a prolonged antifungal drug treatment, mice have more severe colitis and a significant change in composition of their fungal microbiota [86]. Recently, studies found that the gastrointestinal microbiota and the interplay between the host immune system and the gut flora may be associated with IBD onset and exacerbation [87]. A polymorphic variant of Dectin-1 is related to IBD severity in patients [8]. Moreover, mice lacking Dectin-1 are more susceptible to colitis. *Candida* *tropicalis* contribute to a more severe colitis pathology in mice with Dectin-1 deficiency than wild-type mice. An increased production of IL-17 and IFN-γ by T cells along with increased TNF-α, IL-23p19, IL-17a and defensins detected from the colons of Dectin-1^–^^/^^–^ mice. The observation of the increased production of inflammatory mediators along with the more severe colitis in Dectin-1^–^^/^^–^ mice suggests an inability of Dectin-1 deficiency to activate effective immune responses to a specific pathogen, which promotes the inflammation and leads to more severe colitis [88]. This research suggests a role played by fungi to stimulate immune responses in the gut through Dectin-1 in the pathology of IBD. It is more interesting that Dectin-1 is also involved in aggravating inflammation despite its protective role against fungi pathogen. A lack of Dectin-1 signaling can facilitate the growth and expansion of *Lactobacilli*, which exerts protective effects during colitis. However, which role will Dectin-1 play depends on the situation of the gut microbiota. Overall, Dectin-1 can serve as a double-edged sword with a positive effect of protecting the host against the infection as well as a negative effect of hindering the expansion of beneficial bacteria [89, 90]. Besides Dectin-1, Dectin-3 was also involved in the pathogenesis of IBD. Mice lacking Dectin-3 are more susceptible to colitis and have higher fungal burden in the gut. Furthermore, supplemental of *C. tropicalis* promoted the pathogenesis of colitis in Dectin-3-deficiency mice [46, 91].

Cancer is associated with changes in the interactions between the gut microbiota, intestinal epithelium and host immune system [92]. Colorectal carcinogenesis can be affected by the gut flora through various mechanisms including specific alterations in the composition of gut microbiota and by-products that target IECs [93]. A study characterizing the mycobiotic signatures of 131 subjects, which were divided into polyp and colorectal-cancer (CRC) groups, along with a healthy control population for observing the fungal dysbiosis, found decreased fungal diversity. An increased *Ascomycota/Basidiomycota* ratio along with an increased growth of *Trichosporon* and *Malassezia* was also recognized. This alteration indicates that fungal dysbiosis and its related immune responses may be involved in polyp and CRC pathogenesis [94]. In patients with colorectal adenoma, there is also a change in the fungal microbiota structure at different stages of adenoma [95]. Recently, CARD9 deficiency was found to induce myeloid cell differentiation into myeloid-derived suppressor cells with increased *C. tropicalis*, which finally promotes the progression of colon cancer [96, 97]. Although the role of fungi in the pathogenesis of CRC gradually came into view, how CLRs participate in this process remains unclear to us. One study found that Dectin-1 expression is up-regulated in hepatic fibrosis and liver cancer, and Dectin-1 protects against inflammation-induced oncogenesis by suppressing TLR4 signaling [18]. Furthermore, Dectin-1 expressed on DCs and macrophages is critical in the recognition of and signaling by tumor cells [98]. In summary, CLRs are the predominant PRRs involved in recognition of the mycobiota and regulation of gastrointestinal immunity. Important roles can be played by CLRs in the development and progression of gastrointestinal illness, and much could be gained by exploring therapeutic approaches for mycobiota-related diseases.

## Future outlook

In this review, we reflected on recent studies on the roles of CLRs in the gastrointestinal environment and how CLRs interact with the immune system, as well as on diseases related to these processes. The harmonious symbiosis, which is maintained between commensal microorganisms and the host, is critical for good health. Maintenance of this relationship requires CLRs acting as PRRs to recognize potential pathogens to mediate innate and adaptive immune responses, which promote health and ameliorate disease. Future studies will provide more insight into how CLRs are involved in various diseases and how to exploit the interactions between the commensal fungal populations and immune responses in the gut to treat related diseases, including IBD and colon cancer. Furthermore, it remains unclear whether and how host immune responses, which are essential for protecting against serious life-threatening fungal infections, are related to disease progression. More work is needed to elucidate the relationship between the immune system and infectious challenges and to understand what strategies can be devised to treat the related diseases.

## Authors’ contributions

T.T.W., S.N.S. and Y.Y.H. provided the idea and designed the work; T.H.L. wrote and revised the paper; L.L. polished the English language and revised the paper. All authors reviewed and approved the final manuscript.

## Funding

This work is supported by grants from the National Natural Science Foundation of China (81572354 and 81772542 to T.W.) and the Natural Science Foundation of Jiangsu Province in China (BK20161400 to T.W.). 
